# Cardiomyopathy and Death Following Chikungunya Infection: An Increasingly Common Outcome

**DOI:** 10.3390/tropicalmed6030108

**Published:** 2021-06-22

**Authors:** Elizabeth M. Traverse, Hannah K. Hopkins, Vedana Vaidhyanathan, Kelli L. Barr

**Affiliations:** 1 Center for Global Health and Infectious Disease Research, University of South Florida, Tampa, FL 33612, USA; emtraverse@usf.edu (E.M.T.); Hannahhopkins@usf.edu (H.K.H.); 2 Central Libraries Research Engagements, Baylor University, Waco, TX 76706, USA; Vedana_vaidhyanathan@baylor.edu

**Keywords:** CHIKV, chikungunya, myocarditis, cardiomyopathy, cardiovascular

## Abstract

Chikungunya virus (CHIKV) is vectored by *Aedes aegypti* and *Aedes albopictus* mosquitoes and is found throughout tropical and sub-tropical regions. While most infections cause mild symptoms such as fever and arthralgia, there have been cases in which cardiac involvement has been reported. In adults, case reports include symptoms ranging from tachycardia and arrythmia, to myocarditis and cardiac arrest. In children, case reports describe symptoms such as arrythmia, myocarditis, and heart failure. Case reports of perinatal and neonatal CHIKV infections have also described cardiovascular compromise, including myocardial hypertrophy, ventricular dysfunction, myocarditis, and death. Myocarditis refers to inflammation of the heart tissue, which can be caused by viral infection, thus becoming viral myocarditis. Since viral myocarditis is linked as a causative factor of other cardiomyopathies, including dilated cardiomyopathy, in which the heart muscle weakens and fails to pump blood properly, the connection between CHIKV and the heart is concerning. We searched Pubmed, Embase, LILACS, and Google Scholar to identify case reports of CHIKV infections where cardiac symptoms were reported. We utilized NCBI Virus and NCBI Nucleotide to explore the lineage/evolution of strains associated with these outbreaks. Statistical analysis was performed to identify which clinical features were associated with death. Phylogenetic analysis determined that CHIKV infections with cardiac symptoms are associated with the Asian, the East Central South African, and the Indian Ocean lineages. Of patients admitted to hospital, death rates ranged from 26–48%. Myocarditis, hypertension, pre-existing conditions, and the development of heart failure were significantly correlated with death. As such, clinicians should be aware in their treatment and follow-up of patients.

## 1. Introduction

Chikungunya (CHIKV) is a positive sense RNA alphavirus generally vectored by *Aedes aegypti* and *Aedes albopictus* mosquitoes [1,2]. On the approximately 12 kb RNA genome of CHIKV, there are two open reading frames in which the non-structural proteins nsP1, nsP2 (helicase), nsP3, and nsP4 (polymerase) or the structural proteins E1, E2, and E3 envelope proteins, capsid, and 6k are coded for, respectively [2,3]. After human infection via an infected mosquito’s bite, CHIKV virions can target a vast array of cell types, including endothelial, epithelial, fibroblastic, and even muscle satellite cells [3,4,5]. Within vertebrate cells, the full virion replication cycle is typically complete in 8 h, and a host will generally begin to show symptoms between four and seven days following the initial mosquito bite [3,4].

Originally isolated in Tanzania in 1952, it is suspected that outbreaks of CHIKV may have occurred as early as 1823 in Zanzibar, and kept mostly to Africa and Asia prior to 2007 [1,6]. Phylogenetic analysis breaks CHIKV down into three major branches, the West African lineage, the East, Central, and South African lineage (ECSA), and the Asian lineage [1,7,8]. Between the 1960s and 1980s, several outbreaks caused by these strains occurred across tropical and subtropical regions of Africa and Asia, until an apparent lapse in activity which was resumed in 2004 when an additional phylogenetic group derived from the ECSA strain was identified, the Indian Ocean sub-lineage (IOL) [1,7,9]. Following the CHIKV reemergence in 2004–2005, several new outbreaks began, specifically on La Reunion, which introduced a new strain variant with the E1 A226V mutant [1,2,10]. This mutation is thought to increase transmissibility and infection in *Ae. albopictus* mosquitos [10]. From 2005 and onward, there have been several outbreaks across Africa and Asia, spreading to Italy, South and Central America, and the Caribbean [1]. While many of these outbreaks in the Americas are of the Asian lineage, which have begun to accrue their own mutations and possibly a sub-lineage, ECSA strains are also present, especially in Brazil [8,11,12]. Rezza et al. shows a comprehensive diagram of strains and their spread across the world [13]. Corresponding with the La Reunion (2005–2006) and later outbreaks was an increase in reports of differing and serious clinical presentations of CHIKV infection, including ocular, renal, respiratory, neurological, and cardiovascular complications [1,2,7,10,14,15].

**Figure 1:**
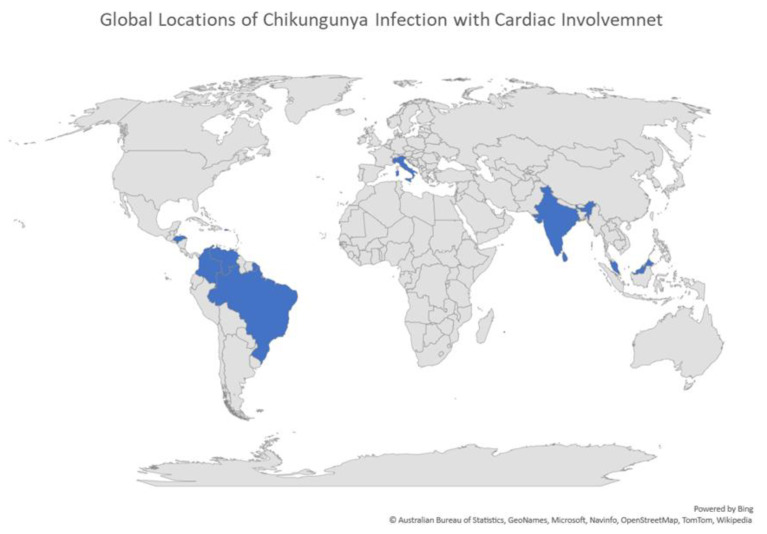
Countries in which a CHIKV infection involved altered cardiac status spanning 1970–2020.

When a human is infected with CHIKV, it usually results in fever, muscle pain, polyarthralgia, and occasionally a rash [3,7]. Recent reports have described cardiac involvement during infections across the world (Table 1 and Figure 1). These cases occur in all age groups, from neonates to the elderly, and generally those who are older and with cardiac related preexisting conditions seem to be at higher risk [16,17]. Many of the cardiac symptoms include arrythmias, abnormal echocardiograms and electrocardiograms, myocarditis and death [15]. Further, the finding of viral myocarditis is concerning due to its connection with other cardiomyopathies such as dilated cardiomyopathy, which can also result in death [18,19]. While most CHIKV infections result in mild symptoms, myocarditis does not always present overtly, which could lead to a future high burden on healthcare systems where outbreaks have occurred [18]; a number of these countries have underdeveloped infrastructure; thus, this burden could be overwhelming [20,21]. Since there is no distinct course of action for CHIKV infection, nor a vaccine, CHIKV infection should be concerning to clinicians [22]. 

## 2. Materials and Methods

Case study literature included in this review was sourced from the following databases: Pubmed, Embase, LILACS, and Google Scholar. The following search terms were used: “(‘CHIKV’/exp OR CHIKV) AND (‘heart disease’/exp OR ‘heart disease’)”, “CHIKV and heart”, “CHIKV and myocarditis”, “CHIKV virus infection and cardiovascular involvement”. Some articles were found via direct search based on works cited by other articles. Inclusion criteria for case studies included confirmed CHIKV infection and altered cardiac status, including reported tachycardia, bradycardia, arrythmia, myocarditis, myocardial infarct, abnormal electrocardiogram, abnormal echocardiogram, heart failure, cardiac arrest, and/or cardiorespiratory failure. Case reports of individuals that were pregnant but not in the peri-partum period, and those with malaria or autoimmune conditions (i.e., lupus, HIV) were excluded from the analysis. Statistical tests included ANOVA, Student’s *t*-test, and Pearson’s correlation tests.

**Figure 2:**
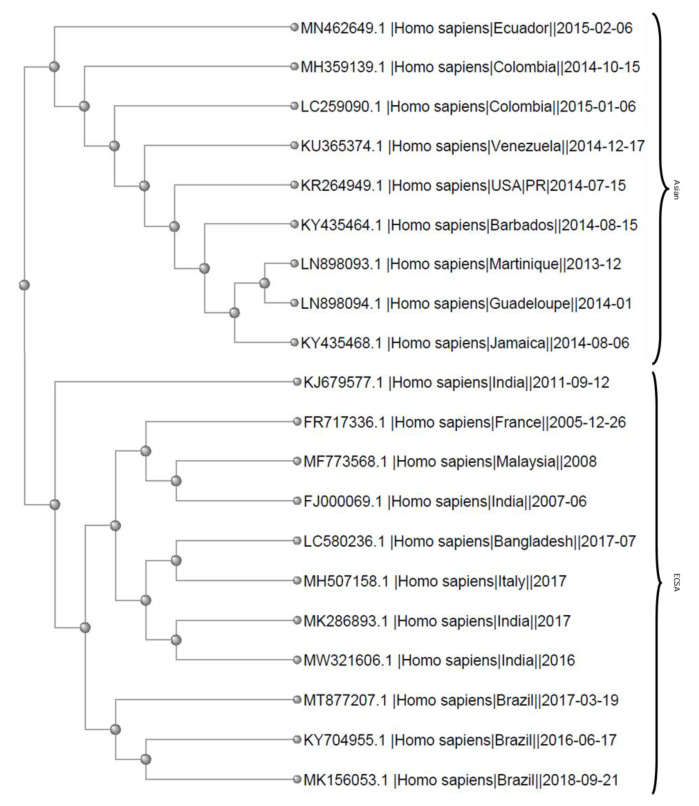
Phylogenetic tree showing strains of CHIKV taken from *Homo sapiens* samples that were obtained at the same location during the outbreaks in which altered cardiac status was reported. The strains and tree were developed using the NCBI viruses software [23].

**Figure 3:**
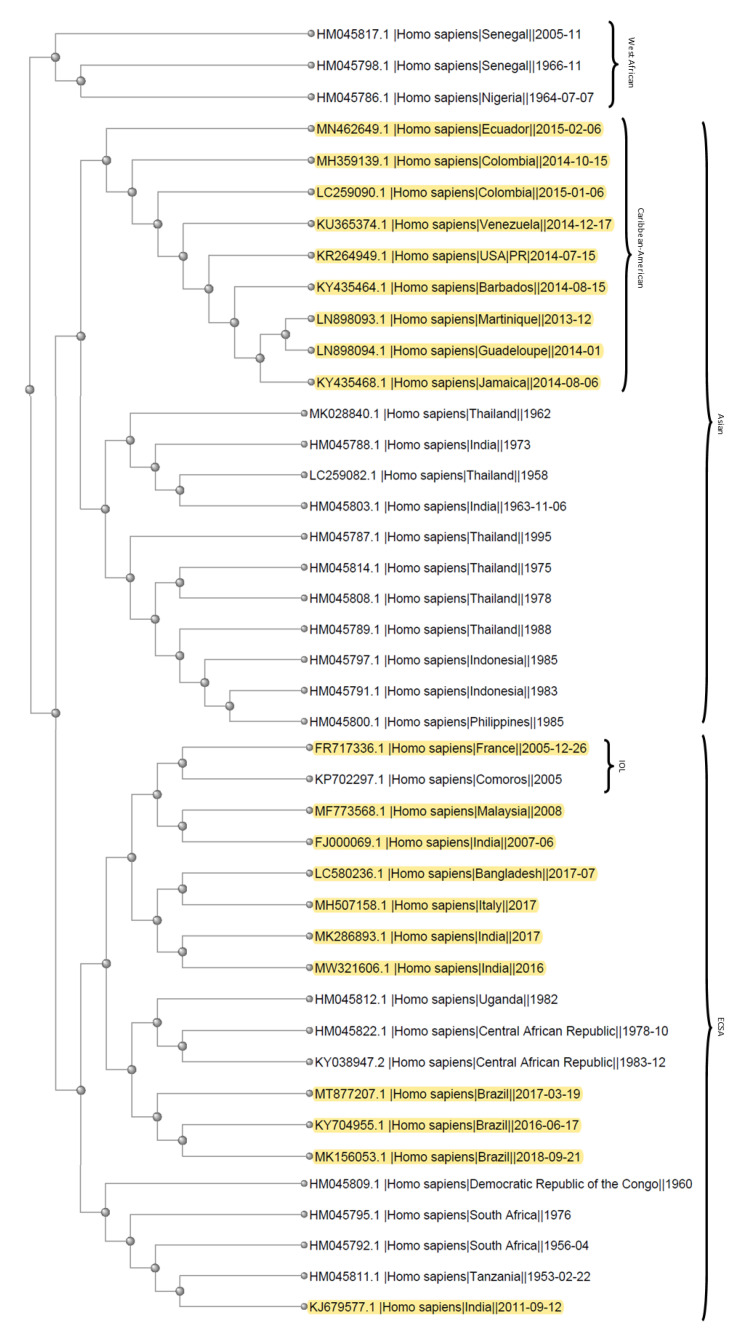
Phylogenetic tree listing several outbreaks from 1953 through 2005, with the addition of the strains in Figure 2. These strains and the tree were developed using the NCBI Viruses database [23]. Based on the strain data from NCBI, the three main branches are labeled to indicate West African, ECSA, or Asian lineages. Additionally, the sub-lineages for IOL and Caribbean-American are labeled. The strains from Figure 2 are highlighted.

Phylogenetic trees were created using the data and phylogenetic analysis software on NCBI Virus (https://www.ncbi.nlm.nih.gov/labs/virus/vssi/#/ (accessed on 21 June 2021)) [23]. Data points were selected based on year and location in which the samples were collected, exclusively from *Homo sapiens* samples. Sequences used were at least 10,000 nucleotides long, in an effort to get as close to the full sequence as possible. Specifically, data points for Figure 2 were selected based on the collection date corresponding to year and location in which a CHIKV outbreak had at least one reported case of altered cardiac status (see Table 1). For Figure 3, the same data points as Figure 2 (highlighted) were expanded upon to include data from all outbreaks through 2005 that fell under the criteria described above. For both trees, only one sample from each year and location combination was included. 

## 3. Results

### 3.1. Locations of CHIKV Infections with Cardiac Symptoms

CHIKV infections with cardiac symptoms were reported across the globe (Figure 1). The earliest of these reports occurred in Sri Lanka and India in the 1970s, followed by a gap of nearly 30 years without report, which likely coincided with a reduction in CHIKV outbreaks [1,24,25,26] (Table 1). Following the Reunion Island outbreak in 2004–2005, an increase in reporting of cardiac related incidents during CHIKV infection occurred, which may indicate newer strains have a higher likelihood of affecting the heart, or a lack of reporting in previous outbreaks [1,11,27] (Table 1).

### 3.2. Phylogenetics

Phylogenetic trees were designed using the NCBI Viruses software [23]. Analysis of CHIKV taken from human patients that correlate with outbreaks in which altered cardiac status was reported produced two major branches, representing the Asian and ESCA strains which have spread throughout the world in a documented manner (Figure 2) [13]. There were no reports of the West African lineage causing cardiac related symptoms.

Analysis of CHIKV taken from human patients that correlate with outbreaks in which altered cardiac status and 22 additional sequences from other CHIKV outbreaks (1953–2005) was performed in order to provide additional genetic material to determine if the bifurcated tree in Figure 2 was significant [23]. The analysis shows three major branches, representing the West African, ECSA, and Asian lineages (Figure 3). Again, no reports of cardiac involvement were associated with the West African lineage.

### 3.3. CHIKV Causes Cardiac Symptoms and Death

In non-pregnant adults (those over 18 years), there was a range of altered cardiac status. Many sources reported arrythmia in general, as well as palpitations [15,46,53,76,77] (Table 2 and Table 3). Additional cardiac alterations included myocardial infarct, heart failure, cardiac arrest, and myocarditis [14,26,29,30,41,43,44,53,61,67,74] (Table 2 and Table 3). In children (ages 1 year to 18 years), the symptoms were similar to that in adults with tachycardia, bradycardia, hypertensive shock, myocarditis, cardiorespiratory failure, and death being reported [2,34,38,50,52,66,72] (Table 2 and Table 3). Reports of altered cardiac symptoms in infants include tachycardia, heart murmur, hypertension, ventricular dysfunction, pericardial effusion, myocarditis, and death [32,33,42,45,51,54,57,58,60,63,69,78] (Table 2 and Table 3). Pregnant mothers were reported to experience gestational hypertension, cardiorespiratory failure, and death [62,69] (Table 2 and Table 3).

There are a number of cases in which the CHIKV infection may have aggravated a pre-existing heart condition, including hypertension, ischemic heart disease, cardiac compromise, and heart failure [37,41,44,55,61,70,71,74,79,80] (Table 2 and Table 3). Additionally, those who are elderly, especially if they have cardiac alterations, were found to be at higher risk of death [81] (Table 2 and Table 3). However, there are also reports of individuals with no pre-existing conditions dying of acute cardiac arrest, cardiorespiratory failure, and cardiac decompensation, including infants and children following CHIKV infection [57,60,66,69] (Table 2 and Table 3).

In three studies, patients who developed myocarditis, with or without pre-existing conditions, were at risk of death [44,52,57] (Table 2 and Table 3). Five studies reported that when patients were admitted to hospitals, the mortality rate for CHIKV positive patients with severe conditions (including cardiac compromise) ranged from 26% to 48% [30,43,44,49,82] (Table 2 and Table 3). In order to explore specific clinical features and their correlation with death, studies were divided into single patient case reports and clinical studies with multiple patients. It is of note that all of the cases found were of the Asian and ECSA lineages and sub-lineages. Data showing altered cardiac status was not found in the West African strain.

### 3.4. Clinical Features of Single Patient Case Reports

Of 23 unique case reports, 11 were male, 10 were female and two were not reported (Table 2 and Table 4). Seven cases had documented pre-existing conditions (Table 2 and Table 4). Tachycardia was documented in nine cases, arrythmia in 12 but no cases reported palpitations (Table 2 and Table 4). Six patients had abnormal electrocardiograms and seven had abnormal echocardiograms (Table 2 and Table 4). Four patients had clinical hypertension while five developed hypotension (Table 2 and Table 4). Cardiomegaly was observed in four cases and heart failure reported in 11 cases (Table 2 and Table 4). Myocarditis was reported in four cases (Table 2 and Table 4).

Death caused directly by CHIKV infection is not commonly reported; however, there are reports of excess deaths in countries in which a CHIKV epidemics have occurred [20,21,55,87,88] (Table 2 and Table 4). In regard to CHIKV infections with altered cardiac status, 11 deaths were reported out of 23 individual case reports (48%) (Table 4). There was no correlation between the incidence of pre-existing conditions, hypertension, hypotension, abnormal electrocardiogram/echocardiogram, palpitations, arrythmias, tachycardia, A226V mutation, sex, or cardiomegaly with death (Table 4). However, patients infected with the Asian lineage and those with heart failure or myocarditis were significantly correlated with death (Table 4) (*p* = 0.0224, *p* = 0.0213, *p* = 0.036).

### 3.5. Clinical Features of Patients from Clinical Studies with Multiple Patients

We identified 17 studies that described 2880 patients in clinical studies combining data from multiple patients. When complied, the data show that men and women were equally represented for having cardiac symptoms (*p* = 0.2379, *p* = 0.0623, respectively) (Table 5). Nor was there a specific CHIKV lineage associated with death (*p* = 0.2534) (Table 5). The most commonly reported clinical features were having a pre-existing condition and hypertension (Table 5). Electrocardiograms and echocardiograms were performed in a fraction of patients. When reported, 95% of CHIKV-infected patients had abnormal electrocardiogram and 82% had abnormal echocardiograms (Table 5); 142 (5%) of 2880 patients died (Table 5). Here death was significantly correlated with having a pre-existing condition (*p* = 0.0001), having hypertension (*p* = 0.0001), and having heart failure (*p* = 0.0001) (Table 5).

## 4. Discussion

Based on geographic location, the areas most affected by heart complications during CHIKV infection are India, portions of South America, and the Caribbean (Figure 1). Upon initial inspection, it might appear that these complications are related to the Asian lineage, due to these areas reported coinciding with Asian CHIKV circulation, especially those in South America and the Caribbean [1,8,11,13,27]. However, upon further exploration there was not a specific lineage connected to cardiac symptoms (Table 1).

Interestingly, there were no reports of the West African lineage causing cardiac related symptoms. This may imply that the 15–22% nucleotide difference between the West African strains and the ECSA and Asian lineages are associated with cardiac symptom abnormalities [89,90]. Further, due to our analysis, there is no particular lineage or mutation, such as the A226V mutation, that increases risk of heart complications during CHIKV infection. While the majority of reported cases occur around 2005 and later, which might also implicate the A226V mutation, the cases reported in the 1970s and the clustering evident in the phylogenetic trees and statistical analysis indicate that the A226V mutation is not associated with cardiac involvement [1,2,7,10,14,15,24,25]. This is supported by the number of reports from countries in South America and the Caribbean, where the circulating CHIKV are all derived from the Asian lineage and clustered together on the tree (Figure 2 and Figure 3). This clade of related strains has been referred to as the Caribbean or Caribbean-American clade [11,27]. Based on reports of the CHIKV outbreaks in these areas, there is a wide range of non-synonymous mutations, none of which are found in all or the majority of cases [1,11,27].

Arrythmias were a consistent symptom, as patients presented with tachycardia, the heart beating faster than the average of 60–100 bpm [46,67,91,92]. While this is not a definitive confirmation of cardiac distress, some patients received electrocardiograms, showing repolarization disturbances, poor R wave progression, and/or right bundle branch block [67,71,85]. Repolarization disturbances refers to alterations in the charging to membrane potential in the heart, R wave progression refers to the QRS complex, and the right bundle branch block refers to clotting [93,94]. Some patients received echocardiograms, showing altered left ventricular (LV) ejection fraction, hypo-contractility, and/or dilated LV cardiomyopathy [67,68,71]. Ejection fraction measures contractile dysfunction [95]. Interestingly, some patients presented with bradycardia, when the heart rate drops below 60 bpm [46,47,48]. Some of these patients had to be taken off their medications for hypertension in order to prevent hypotensive crisis [47]. Death can be caused by any of these conditions, risk factors for which is older age and cardiac comorbidities such as hypertension and congestive heart failure [29,31,43,47,70,74,86,96,97]. Children usually do not have comorbidities; however, in one case there was an individual with a mutation of the SCN5A gene, which is linked to cardiac sinus node dysfunction [84]. This 10 year old presented with CHIKV infection and bradycardia, and later a pace maker had to be installed [84]. This finding is in line with that in adults; cardiac related comorbidities can be exacerbated by CHIKV infection.

One symptom of CHIKV infection that is found in all age groups has been myocarditis. Myocarditis refers to inflammation of heart tissue, and it can be caused by viral infection, thus becoming viral myocarditis [98]. For viral myocarditis to occur, the cardiomyocytes must be directly infected, resulting in cell death [19,98]. CHIKV has a broad tropism, and there is evidence for cardiac infection such as receptors for CHIKV on cardiomyocytes, including MXRA8 (low expression) and PBH (medium expression), as well as evidence of viral antigen in cardiac tissues [8,53,99,100,101]. Further, some studies indicate that depending on viral titer, the damage caused can be worse, though this was conducted in animal models and more work must be done to find if different viral strains in humans cause different titers and thus worse damage [102,103,104]. The damage induces the innate immune response, including the recruitment of natural killer cells (NKCs) and the release of cytokines, gamma interferon, and nitric oxide [18,19,98]. While NKCs and some cytokines can have a cardioprotective effect, other cytokines and players in the adaptive immune response, specifically T-lymphocytes, can cause further damage by killing more cardiomyocytes [18,19,98]. Following this, fibrosis begins to replace the damaged or dead cardiomyocytes [19,98]. Fibrotic infiltration of cardiac tissue is a keystone of another disease, dilated cardiomyopathy (DCM) [98,105]. DCM usually presents with left ventricular (LV) dysfunction, as it dilates and the heart wall thins causing contractility issues [106]. DCM can lead to heart failure [106]. Considering how prevalent viral myocarditis can be among all age groups during CHIKV infection, the danger of later DCM diagnosis for these patients is concerning. Additionally, viral myocarditis has other complications associated with it, including arrythmia, myocardial infarction, and sudden cardiac death [107]. In children, myocarditis is most commonly caused by viral infection [108], though reports show that many children later recover and/or only have mild symptoms [50,108]. Individuals who are infected by CHIKV should have their heart condition monitored via echocardiogram and/or electrocardiogram from time of hospital admittance and beyond for possible altered cardiac status so physicians may intercede if complications ensue.

Of patients admitted to hospitals, the mortality rate for CHIKV positive patients with severe conditions (including cardiac compromise) ranges from 26% to 48% [30,43,44,49,82]. While there are few studies that thoroughly examine the number of CHIKV patients who present with cardiac complications (including the use of echocardiograms to verify arrythmias), Castillo et al. estimates that 10% of their 32 patients exhibited heart complications [76]. If this is representative of all CHIKV infections, and of those cases 26–48% prove fatal, there is an unreported burden to the many countries where CHIKV circulates.

CHIKV co-circulates with other arboviruses such as Dengue virus and Zika virus, and coinfection of these diseases can cause worsened symptoms [2,15,109,110]. In fact, there are reports of Dengue and Zika induced viral myocarditis, and reports that a prior arbovirus infection can be an additional risk factor for myocarditis [15,24,25,109,110]. Clinicians should ensure taking an accurate history of patients to mitigate any additional cardiovascular risks caused by coinfections or previous exposures.

## 5. Conclusions

There is limited or inaccurate reporting in many of the areas where CHIKV infections have surfaced, frequently due to lack of infrastructure [20,21]. It is possible that there are additional cardiac symptoms of CHIKV infection that are unknown, or additional patients that did not seek medical care due to access issues. Further, many CHIKV infections are self-limited and mild such that, even if viral myocarditis is present, the patient may not show overt symptoms prompting them to seek medical care [98]. All these factors present a clinical challenge. Physicians have recommended the use of electrocardiograms and/or echocardiograms on patients at risk of CHIKV infection, especially patients with risk factors such as old age and cardio-related comorbidities [81]. This would allow for detection of some of the more dangerous symptoms, such as myocarditis [107,111]. Additionally, history of CHIKV infection should be considered for patients presenting with early forms of DCM considering the connection between myocarditis and cardiomyopathies [18,98]. Combined, these clinical aspects of CHIKV outbreaks may be able to alleviate future ramifications on the health-care system as people acquire cardiomyopathy later in life.

In children specifically, it has been shown that myocarditis can cause death and incomplete recovery, and can require heart transplants [112]. There is no vaccine or specific treatment for CHIKV infection, though there is work ongoing in animal models [22,113]. Further, the knowledge of CHIKV infection among some health-care providers is lacking [114]. While the incidence of altered cardiac status due to CHIKV infection appears to be low, its true burden is unknown, and it is a risk for anyone with comorbidities such as hypertension. CHIKV should no longer be treated as a self-limited febrile illness; the data clearly show that CHIKV can kill.

## Figures and Tables

**Table 1:** Location and year of case studies in which altered cardiac status was reported. Strain information was taken from these case studies when available or taken from other sources reporting outbreaks in the same locations in the same year.

Location	Year	Lineage
Sri Lanka [24,25]	1972	Asian [28]
India [26]	1978	Asian [28]
Reunion Island [29,30,31,32,33,34]	2005	IOL [35]
Kerala, India [36]	2007	IOL [36]
Malaysia [37]	2008	ECSA [37]
Aaryad, Kerala, India [38]	2009	IOL [39,40]
Malaysia [41]	2010	ECSA [41]
India [42]	2011	ECSA [39]
Guadeloupe and Martinique [43]	2013–2014	Asian [27]
Caribbean [44]	2013–2014	Asian [27]
Jamaica [45]	2014	Asian [27]
Venezuela [46,47,48,49]	2014	Asian [49]
Barbados [50]	2014	Asian [50]
French West Indies [51]	2014	Asian [51]
Tahiti, French Polynesia [52]	2014	Asian [52]
Puerto Rico [53,54,55]	2014	Asian [56]
San Salvador/El Salvador [57]	2014	Asian [57]
Willemstad, Curacao [58]	2014	Asian [59]
French Guiana [14]	2014–2015	Asian [11]
Sucre, Colombia [60]	2014–2015	Asian [11]
Columbia [61]	2015	Asian [11]
Machala, Ecuador [62]	2015	Asian [62]
North India [63]	2016	Asian or ECSA [64]
Paraiba, Brazil [65]	2016	Asian or ECSA [12]
Ceara, Brazil [66]	2016–2017	Asian or ECSA [12]
Brazil [21,67,68,69]	2017	Asian or ECSA [12]
Dhaka, Bangladesh [70]	2017	IOL [70]
Italy [71]	2017	IOL [71]
India [72]	2017	ECSA [73]
Columbia [74]	2018	Asian [11]
Brazil [75]	2018	Asian or ECSA [12]

**Table 2:** Cardiac complications from individual patients infected with CHIKV. Lineage was determined from the data in the case studies when available. When the lineage was not reported, the year and location was matched to isolates in NCBI Nucleotide with Table 1 to deduce the lineage. NR = not reported, CABG = coronary artery bypass graft.

Patient	Sex/Age	Pre-Existing Conditions	Symptoms	Lineage
1	F/44		Tachycardia, ventricular abnormalities, gallop rhythm, cardiomegaly, abnormal electrocardiogram, later developed congestive cardiomyopathy [24]	Asian
2	M/46	Fever three months prior	Atrial fibrillation and cardiomegaly [24]	Asian
3	F/21		Myopericarditis, pericardial effusion, abnormal echocardiogram, abnormal electrocardiogram [83]	IOL
4	M/5.5		Myocarditis, abnormal electrocardiogram, abnormal echocardiogram, congestive cardiac failure [38]	IOL
5	M/71	Hypertension, alcoholic cardiomyopathy	Acute decompensated heart failure [75]	ECSA or Asian
6	M/28		Tachycardia, hypotension, abnormal electrocardiogram, abnormal echocardiogram, pericardial effusion, altered LV ejection fraction, LV hypercontractility, myocarditis, DENV coinfection [67]	ECSA or Asian
7	M/77	Hypertension, ischemic heart disease, CABG	Acute cardiac arrest, death, abnormal electrocardiogram, abnormal echocardiogram, dilated left cardiomyopathy [71]	IOL
8	M/0		Tachycardia, bradycardia, pulmonary hemorrhage, death [69]	ECSA or Asian
9	NR		Fetal heart rhythm abnormalities, fetal distress, intracerebral hemorrhage, death [58]	Asian
10	NR		Fetal heart rate abnormalities, fetal distress, skin rash went away [58]	Asian
11	M/12		Hypotensive shock, hemodynamic instability, abnormal echocardiogram, LV hypokinesia, LV dysfunction, hypotension, myocarditis [72]	ECSA
12	M/5		Cardiorespiratory arrest, death [66]	ECSA or Asian
13	F/51		Respiratory difficulties, cardiorespiratory arrest, death [66]	ECSA or Asian
14	F/0.25		Tachycardia, abnormal electrocardiogram [78]	ECSA or Asian
15	F/0		Fetal pericardial effusion, cardiomegaly [42]	ECSA or Asian
16	M/75		Tachycardia, multi-organ failure, hemodynamic instability, atrial fibrillation, cardiorespiratory arrest, death [49]	Asian
17	M/65	Hypertension	Tachycardia, hypotensive, cardiac arrest, death [49]	Asian
18	F/32		Hypotension, tachycardia, hemodynamic instability, abnormal echocardiogram [49]	Asian
19	F/10	SCN5A mutation, bradycardia	Bradycardia, cardiac sinus node dysfunction, needed pacemaker [84]	IOL
20	F/66		Tachycardia, became hypertensive, cardiac decompensation, cardiac arrhythmia, death [41]	ECSA
21	F/0		Bradycardia, hypotensive, possible septic shock, death [45]	Asian
22	M/87	DENV, leptospirosis, chronic heart failure	Cardiac deterioration, death [74]	Asian
23	F/53	Hypertension, cardiac failure	Cardiomegaly, abnormal echocardiogram, death [37]	ECSA

**Table 3:** Cardiac complications from literature that complied multiple patients. Lineage was obtained from the data when available. When unavailable, the year and location was matched to isolates in NCBI Nucleotide with Table 1 to deduce the lineage.

Total Patients	# Male	# Female	Age Range	Pre-Existing Conditions	Symptoms	Lineage
42	20	22	Median 60, 17 over 65, 16 under 40		All patients showed palpitations, abnormal electrocardiogram in 71.4%, myocarditis suspected in 100% [85]	Asian
16	14	2	Median 9.5 days, up to 3 months		Cardiovascular involvement including high pulmonary pressures in 4, myocarditis in 1, one death due to multi-organ failure [50]	Asian
399 confirmed, 291 probables	395	295	Median 38 years, range 21–96	8.9% with hypertension, 1.5% with ischemic heart disease	Hypotension in 5, mortality rate of 0.5% [70]	IOL
21	10	11	Median 3 months		At least 1 with myocarditis [54]	Asian
64	37	27	Median 62, range 49–71	Hypertension in 37, chronic heart failure in 12	2 had myocarditis, evidence of exacerbation of preexisting conditions, both myocarditis patients died (they did not have preexisting conditions, 11 and 56/F) [52]	Asian
203 suspected, 69 confirmed			Children less than 15 years		DENV coinfection, myocarditis, and bradycardia in one [50]	Asian
287	117	170	59 +/− 8		91 had palpitations, 45% had arrythmia (33% bradyarrhythmia), 19 cases of tachyarrhythmia, tachycardia, 3 cases of sudden death. Possible myocarditis [48]	Asian
180	167	13	68.8 +/− 16.2, 54.8 +/− 16.5	Hypertension in 118, coronary heart disease in 32, congestive heart failure in 13	4 patients experienced exacerbation of congestive heart failure [86]	Asian
257	133	124	63 +/− 9	Hypertension in 63	Arterial hypotension in 25, 51% had arrhythmias, 33% had bradyarrhythmia’s [47]	Asian
43	18	25	mean 62.5, range 24–88	84% had comorbidities	Chronic cardiac failure in 7, cardiac arrest in 4, related to comorbidities, 49% mortality rate, exacerbation of previous conditions [29]	IOL
209	65	144	99 aged 20–40, 77 aged 40–60, 17 aged 60–80, 13 aged 10–20, 3 aged 80+		Myocarditis in 1 [36]	IOL
610	271	339	median 70, range 15–95	546 with comorbidity	226 with cardiac abnormality, 110 of these had underlying cardiac condition, 137 had hypertension; 84 cases had heart failure, 29 of which had underlying cardiomyopathy, 10 with coronary artery disease, 6 with valvular disease, 4 with history of myocardial infarction, and 1 with arrhythmias; 35 cases of myocarditis, 4 cases of acute myocardial infarction [31]	IOL
33			adults	18% had exacerbations of previous conditions	1 with myocarditis [30]	IOL
38			neonates (average symptoms day 4)		Abnormal echocardiograms in 16, showing hypertrophy in 5, ventricular dysfunction in 2, pericarditis in 2, coronary artery dilation in 6 [32]	IOL
86	50	36	median 3.5, range 3 weeks–17 years		7 total with cardiac symptoms, 2 with heart rhythm disturbances [33]	IOL
9			children		5 with cardiac complications including myocarditis and hemodynamic disorders [34]	IOL
23	19	4	1 aged 31–40, 1 aged 41–50, 4 aged 51–60, 5 aged 61–70, 9 aged 71–80, 3 aged 81–90		3 patients with cardiac involvement, cardiogenic shock causing death in 1 [82]	IOL

**Table 4:** Incidence of specific features of CHIKV-infected patients with cardiac symptoms. Pearson’s Correlation Coefficients were obtained to determine if there were any specific features associated with death.

Feature	N (%)	Correlation Coefficient	*p* Value
Male	11 (48%)	0.1455	0.5293
Female	10 (43%)	−0.1455	0.5293
Preexisting Condition	7 (30%)	0.1234	0.5749
A226V mutation	4 (17%)	−0.2097	0.3370
Asian Lineage	7 (41%)	0.5494	0.0224
Tachycardia	9 (39%)	−0.0542	0.8057
Arrythmia	12 (52%)	0.0454	0.8368
Palpitations	0	NA	NA
Hypertension	4 (17%)	0.2496	0.2507
Hypotension	5 (22%)	−0.0825	0.7080
Myocarditis	4 (17%)	−0.4393	0.0360
Cardiomegaly	4 (17%)	−0.2097	0.3370
Heart Failure	11 (48%)	0.4773	0.0213
Abnormal electrocardiogram	6 (100%)	0	1
Abnormal echocardiogram	7 (100%)	0	1
Death	11 (48%)	NA	NA

**Table 5:** Incidence of specific features of CHIKV-infected patients from clinical studies with cardiac symptoms. Pearson’s Correlation Coefficients were obtained to determine if there were any specific features associated with death.

Feature	N (%)	Correlation Coefficient	*p* Value
Male	1316 (52%)	0.3522	0.2379
Female	1212 (48%)	0.5303	0.0623
Preexisting Condition	935 (32%)	0.8893	0.0001
A226V mutation	9 (53%)	0.2932	0.2534
Asian	8 (47%)	−0.2932	0.2534
IOL	9 (53%)	0.2932	0.2534
Tachycardia	9 (1%)	−0.09995	0.7027
Arrythmia	292 (10%)	0.07249	0.7822
Palpitations	133 (5%)	−0.1372	0.5994
Hypertension	636 (22%)	0.8288	0.0001
Hypotension	37 (1%)	−0.1675	0.5204
Myocarditis	98 (3%)	0.4798	0.0513
Cardiomegaly	0	NA	NA
Heart Failure	116 (4%)	0.9417	0.0001
Abnormal electrocardiogram	35 (95%)	−0.6378	0.2470
Abnormal echocardiogram	18 (82%)	−0.2487	0.6347
Death	142 (5%)	NA	NA

## Data Availability

All data are included in the manuscript.
